# Effect of Pretreatment Methods on the Formation of Advanced Glycation End Products in Fried Shrimp

**DOI:** 10.3390/foods12234362

**Published:** 2023-12-03

**Authors:** Runlin Wu, Caihua Jia, Jianhua Rong, Shanbai Xiong, Ru Liu

**Affiliations:** 1 College of Food Science and Technology, Huazhong Agricultural University, Wuhan 430070, China; wurunlin2018@163.com (R.W.); chjia@mail.hzau.edu.cn (C.J.); rong@mail.hzau.edu.cn (J.R.); xiongsb@mail.hzau.edu.cn (S.X.); 2 National R&D Branch Center for Conventional Freshwater Fish Processing (Wuhan), Wuhan 430070, China; 3 Key Laboratory of Environment Correlative Dietology, Huazhong Agricultural University, Ministry of Education, Wuhan 430070, China

**Keywords:** frying, pretreatment, pacific white shrimp, advanced glycation end products, lipid oxidation, Maillard reaction

## Abstract

Fried shrimp are popular for their attractive organoleptic and nutritional qualities. However, consumers are more concerned about the safety of fried foods. To investigate the formation of advanced glycation end products (AGEs) in fried shrimp and provide pretreatment guidance for producing low-AGEs fried pacific white shrimp were treated with seven pretreatment methods before frying. The AGEs contents, physicochemical indicators, and their correlations in the fried shrimps’ interior, surface, and batter layer were analyzed. Results indicated that pretreatment methods influenced both Maillard and oxidation reactions by altering the basic compositions, which controlled the formation of AGEs. The highest and lowest AGEs contents were obtained in shelled shrimp with exscinded back and whole shrimp, respectively. The batter-coated treatment reduced the AGEs contents in samples but increased the oil content. Correlation analysis showed that lipid oxidation was the decisive chemical reaction to the formation of AGEs by promoting the generation of dicarbonyl compounds and their combination with free amino acids. Conclusively, the whole shrimp was suitable for producing fried shrimp with low AGEs, oil content, and desirable color.

## 1. Introduction

The Pacific white shrimp (*Litopenaeus vannamei*) is one of the largest aquaculture shrimp productions and is well known for its high nutritional qualities (such as protein, polyunsaturated fatty acids, and astaxanthin) and economic value [1]. Frying, microwaving, baking, steaming, and drying are common cooking methods for shrimp [2]. Fried shrimp are popular among consumers due to their unique flavor and texture derived from physicochemical reactions during frying [3,4]. However, these reactions also destroy the nutrients in the fried shrimps, resulting in the oxidative loss of protein, polyunsaturated fatty acids, and astaxanthin [2,5]. Moreover, undesired food hazard factors such as advanced glycation end products, acrylamide, heterocyclic amines, and trans fatty acids, along with the high oil content in fried foods, are harmful to human health, which has raised widespread attention from researchers in recent years [6,7,8].

Numerous studies have shown that foods rich in lipid and protein are prone to producing advanced glycation end products (AGEs) during heating [7,9,10]. In addition, high levels of AGEs content have been detected in various fried meat products [9,11,12]. It was reported high dietary AGEs in food were closely associated with cardiovascular disease, diabetes, and Alzheimer’s disease [9]. AGEs are considered to be a heterogeneous group of compounds, such as N^ε^-carboxymethyllysine (CML), pyrraline, pentosidine, N^ε^-carboxyethyllysine (CEL), and N^ε^-(5-hydro-5-methyl-4-imidazolon-2-yl)-ornithine (MG-H1) [13]. Usually, AGEs are formed in different stages of the Maillard reaction [14]. Additionally, lipid oxidation could produce reactive oxygen radicals, inducing more active dicarbonyl compounds generated by the Amadori rearrangement product reaction and lipid peroxidation [15]. Protein-rich shrimp will absorb frying oil during frying providing precursors for the mentioned reactions. In addition, frying is carried out at 150–200 °C, which benefits the Maillard reaction and lipid oxidation. Therefore, it is hypothesized that AGEs could form in fried shrimp. However, previous studies have mainly focused on the oil content and organoleptic properties of fried shrimp. For instance, various batter formulations and hydrocolloid coatings have been applied to reduce fried shrimp’s oil content or lipid oxidation [3,6,16]. As for the formation and control of food hazard factors in shrimp, Pan, Ji, Liu, and He [17] reported that vacuum frying could reduce the oil and acrylamide contents of breaded shrimps compared with traditional frying under three frying temperatures, and the maximum reduction rate of acrylamide levels up to 60%. Additionally, Zhang, Zha, Dong, and Zhao [5] detected the CML content in shrimp patties heated by different cooking methods (boiling, baking, and microwaving), and reported that a baked shrimp patty had the highest CML content due to using the highest temperature (150 °C) during processing. However, limited literature is available on the formation of AGEs in fried shrimp. In this work, several pretreatment methods are applied to shrimp before cooking, such as beheading, removing the shells, and battering [2,6,17]. Furthermore, whether the pretreatment methods modulate the AGEs contents and sensory quality of the final shrimp products is unknown.

To understand the effects of pretreatment methods on the formation of AGEs in fried shrimps, pacific white shrimps were treated with seven pretreatment methods, including whole shrimp, shelled shrimp, shelled shrimp with exscinded back, shrimps with exscinded back, batter-coated shelled shrimp, batter-coated shelled shrimp with exscinded back, and batter-coated shrimp with exscinded back before frying. The AGEs content, compositions, and chemical reactions in the fried shrimps were determined, and their internal connections were also analyzed. The aim was to clarify the decisive chemical reaction in the formation of AGEs and provide pretreatment guidance for producing low-AGEs contents fried shrimp.

## 2. Materials and Methods

### 2.1. Materials and Chemicals

Frozen Pacific white shrimp (23 ± 3 g), wheat flour (Wuhan Yufeng Grain, Oil and Food Industry Co., Ltd., Wuhan, China), corn Starch (Wuhan Yuxinghang Food Co., Ltd., Wuhan, China), baking powder (Shanghai Fengwei Industrial Co., Ltd., Shanghai, China), NaCl (Hubei Salt Industry Group Co., Ltd., Wuhan, China), white breadcrumbs (Wuhan Jinbao Food Co., Ltd., Wuhan, China), and rapeseed oil (Shandong Luhua Group Co., Ltd., Yantai, China) were purchased from Huazhong Agricultural University market (Wuhan, China).

CML, CEL, and MG-H1 standards were obtained from Toronto Research Chemicals Inc. (Toronto, ON, Canada). Methanol, formic acid, and acetonitrile were LC-MS grade and from Fisher Scientific (Fair Lawn, NJ, USA). The other reagents were analytical grade and from Sinopharm Chemical Reagent Co., Ltd. (Shanghai, China).

### 2.2. Preparation of Fried Shrimps

**Figure 1:**
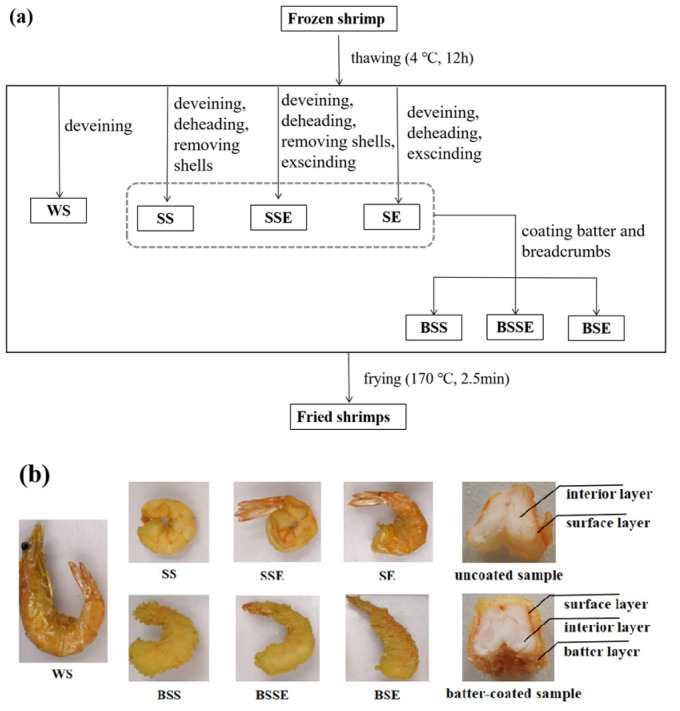
Flow diagram of sample preparation (**a**) and photo of fried shrimps (**b**).

The basic pretreatment process steps of samples are shown in Figure 1a. The details of the seven pretreatment groups are as follows, whole shrimp (WS) only deveined before frying, shelled shrimp (SS) treated with deveining, beheading, removing shells, shelled shrimp with exscinded back (SSE) treated with deveining, beheading, removing shells, exscinding, shrimp with exscinded back (SE) treated with deveining, beheading and exscinding. To obtain the batter-coated shelled shrimp (BSS), batter-coated shelled shrimp with exscinded back (BSSE), and batter-coated shrimp with exscinded back (BSE), the SS, SSE, and SE were coated with batter and breadcrumbs in sequence. The back-exscinded treatment was performed with a knife, a ninety percent-depth slit was exscinded in the abdominal section of the shrimps, and the tails were reserved. The preparation of batter was performed according to the method of Shan et al. [18] with modifications. The basic batter powders comprised wheat flour and corn starch (3:2, *w*/*w*). The weight of baking powder and salt accounted for 0.5% and 1% of the basic batter powders, respectively. All the powders were mixed with water at a ratio of 1:1.1 (*w*/*v*). As for coating shrimp with batter and breadcrumbs, the shaped shrimp were immersed in the batter for 30 s and uniformly coated with breadcrumbs after draining off the excess batter (20 s).

Fresh rapeseed oil (3 L) was preheated to 170 ± 2 °C before frying, and 150 ± 5 g of shrimp were fried for 150 s every time. The excess oil on the fried samples was drained off using filter paper. The fried shrimps (Figure 1b shows the photos of fried shrimps) were divided into the interior, surface (as for samples with shells, the surface layer was the part adjacent to the shell, the depth was about 0.5 mm, and the shrimp shells were discarded) and batter layer (only in the batter-coated treatment groups). To ensure the quality stability of the fried shrimps, the TBARS values and carbonyl content were measured on the day after the preparation of fried shrimps, and the other indicators were determined within one month [7,10]. All samples were uniformly chopped and stored at −20 °C for further analysis.

### 2.3. Measurement of AGEs

The fluorescent AGEs (F-AGEs) content was determined using a F-4600 spectrofluorometer (Shanghai Zhuohao Laboratory Equipment Co., Ltd., Shanghai, China) as described previously [10]. Briefly, samples (2 g) were mixed with phosphate buffer (18 mL, 50 mmol/L, pH 7.4) and subjected to shaking for one hour at 37 °C. Subsequently, the mixtures underwent centrifugation at 5000× *g* for 5 min and filtration. The fluorescence values of the collected filtrates were recorded (ex/em: 345/425 nm). The non-fluorescent AGEs (CML, CEL, and MG-H1) in the fried shrimps were extracted according to the method of Qin et al. [19] with slight modifications. Briefly, freeze-dried samples (100 mg) were treated with chloroform: acetone solution (*v*:*v* = 1:3) in a volume of 3 mL, and the mixtures were centrifuged (4000× *g*, 15 min) for degreasing. The precipitate was redissolved and centrifuged twice. After that, the samples were reduced with 0.4 mL NaBH_4_ in 2 mL borate buffer (0.2 M, pH = 9.2) at 4 °C for 8 h, and then the hydrolysis was performed by adding 5 mL of HCl (6 M, 110 °C, 24 h). A total of 1 mL of hydrolysis solution was dried at 60 °C and re-dissolved with 3 mL of ultrapure water before filtering (nylon 0.22 μm). The filtrate (1 mL) was purified with an MCX cartridge (60 mg/3 mL, Shanghai ANPEL Scientific Instrument Co., Ltd., Shanghai, China), and the target compounds were eluted with 2 mL methanol (containing 5% aqueous ammonia). The collected effluents were dried with nitrogen, reconstituted with ultrapure water (1 mL), and filtered (nylon 0.22 μm) before UPLC-MS/MS analysis. A Waters Acquity UPLC system (Waters Corp., Milford, MA, USA), equipped with a Waters Quattro Micro triple-quadrupole tandem mass spectrometer (MS/MS), was used to quantify non-fluorescent AGEs. The detailed analytical method was reported by Qin et al. [19]. The aliquots (3 μL) were separated using a BEH Amide column (100 × 2.1 mm, 1.7 μm) maintained at 35 °C, and the elution gradients of non-fluorescent AGEs, at a flow rate of 300 μL/min, are shown in Table 1. The source and desolvation temperatures were 110 °C and 350 °C, and the mass spectrometer was operated in positive mode ESI with a capillary voltage of 4 kV; the details of the mass spectrometry parameters are shown in Table 2. Finally, 0.01~0.2 µg/mL of three external standard solutions were used to establish standard curves, and all external standard solutions were treated with solid-phase extraction treatment before UPLC-MS/MS analysis.

### 2.4. Determination of Water, Oil, and Protein Contents

The water, oil, and protein contents were determined according to AOAC official methods 950.46, 991.36, and 928.08, respectively [20]. Briefly, 3.5 g of chopped samples were used to measure water content by gravimetric methods, and 2 g of dry samples were used to determine oil content with Soxhlet apparatus (Alva Instrument Co., Ltd., Jinan, China). In addition, 0.5 g of chopped samples were used to determine protein content with a k980 Kjeldahl nitrogen analyzer (Shanghai Precision Scientific Instrument Co., Ltd., Shanghai, China). The nitrogen-to-protein conversion factor in the non-batter layers was 5.6, while in the batter layer composed of wheat flour and breadcrumbs it was 5.4 [21]. All experiments were performed at least three times.

### 2.5. Determination of Thiobarbituric Acid Reactive Substances (TBARSs)

TBARSs were determined with a N2S spectrophotometer (Shanghai Jingke Industrial Co., Ltd., Shanghai, China) at 532 nm according to Jiang et al. [7]. Samples (3 g) were mixed with 30 mL of trichloroacetic acid and then shaken with a constant temperature shaker (50 °C, 30 min). After cooling, the mixtures were filtered through a double-layer qualitative filter paper. Then, 5 mL of the filtrates were mixed with 5 mL of thiobarbituric acid and reacted for 30 min in a 90 °C water bath. The absorbance of the reaction solution at 532 nm was recorded. 1,1,3,3-tetraethoxypropane standard solutions (0.01–0.25 μg/mL) were used to a obtain a standard curve (y = 1.1927x − 0.0008, R^2^ = 0.9999). The results were expressed as mg malondialdehyde (MAD)/kg sample.

### 2.6. Determination of Carbonyl Content

The extraction of salt-soluble proteins from samples was carried out according to the method of Cofrades et al. [22] with some modifications. Briefly, the chopped samples (2 g) were extracted twice using 20 mL of n-hexane to remove oil, and the defatted samples were mixed with 20 mL of high-salt phosphate buffer (0.02 M, pH 7.0) containing NaH_2_PO_4_, Na_2_HPO_4_ and 0.6 M NaCl. The mixtures were homogenized for 60 s at 4 °C and then centrifuged for 10 min at 10,000× *g* after being stationary for two hours at 4 °C. The concentration of salt-soluble proteins was evaluated using the Lowry method, and the concentration of salt-soluble proteins was calculated according to the standard curve (y = 0.0026x + 0.0155, R^2^ = 0.9961).

The carbonyl content was determined using the method from Mesquita et al. [23] with modifications. Briefly, 2 mL of 2,4-dinitrophenylhydrazine (10 mmol/L in 0.5 mol/L H_3_PO_4_) solution was mixed with 2 mL of protein solutions, and then 1 mL of NaOH (6 mol/L) was added to the mixed solution after 10 min of incubation; the absorbance of the solution was measured at 450 nm after another 10 min of incubation at room temperature. The carbonyl content was calculated using Formula (1).

(1) $$c=\frac{A_{450}\times 10^{9}}{\varepsilon \times b\times C_{pro}}$$

where c is the protein carbonyl of the sample (nmol/mg pro), A_450_ is the absorbance at 450 nm, b is optical path length (1 cm), C_pro_ is protein content (μg/mL), and ε is the molar extinction coefficient (22,308 M^−1^ cm^−1^).

### 2.7. Determination of Free Amino Groups

The content of free amino groups was measured using o-phthaldialdehyde (OPA) reagent according to the method of Sun et al. [24] with slight modifications. Briefly, 10 mL of distilled water was added into freeze-dried samples (200 mg) in a water bath at 95 °C for 15 min. The cooling mixtures were filtered (PES 0.45 μm, Millipore, Merck, Milford) before five-fold dilution. Then, 1.5 mL of OPA reagent was added into 100 μL of dilution solution and incubated for 2 min. Afterward, the absorption was measured immediately with a microplate reader (Multiskan GO, Thermo Fisher, Waltham, MA, USA) at 340 nm. The standard curve was prepared using 0.02–0.1 mg/mL leucine solution (y = 1.8011x − 0.0057, R^2^ = 0.9941), and the results were expressed as mg/g dry sample.

### 2.8. Determination of Browning Intensity

The browning intensity was determined using an N2S spectrophotometer (Shanghai Jingke Industrial Co., Ltd., Shanghai, China) at 420nm according to Jiang et al. [7]. Briefly, samples (2 g) were mixed with 50% ethanol (20 mL), and the mixtures were homogenized for 1 min at 4 °C, and then centrifuged at 11,000× *g* (10 min) after being stationary for 10 min (4 °C). The precipitate was redissolved and centrifuged twice, and the absorbance of supernatants at 420 nm was recorded.

### 2.9. Determination of Color

The color of the shrimps was determined with a Chroma Meter CR-400 (Konica Minolta Inc., Tokyo, Japan) according to the method of Pan, Ji, Liu, and He [17]. As for the surface layer of samples, both sides of the second abdominal segments of the shrimps were measured. After that, the second abdominal segments of samples were cut to obtain the L*, a*, and b* values of the interior of the samples.

### 2.10. Statistical Analysis

All experiments were performed at least three times, and the data were expressed as mean ± standard deviation. The statistical analysis was performed using SPSS 23.0 software (SPSS 23.0, IBM Corp., Armonk, NY, USA). And the significance and correlation analyses were assessed using the Duncan and Pearson’s correlation tests, respectively.

## 3. Results and Discussion

### 3.1. Analysis of AGEs

**Figure 2:**
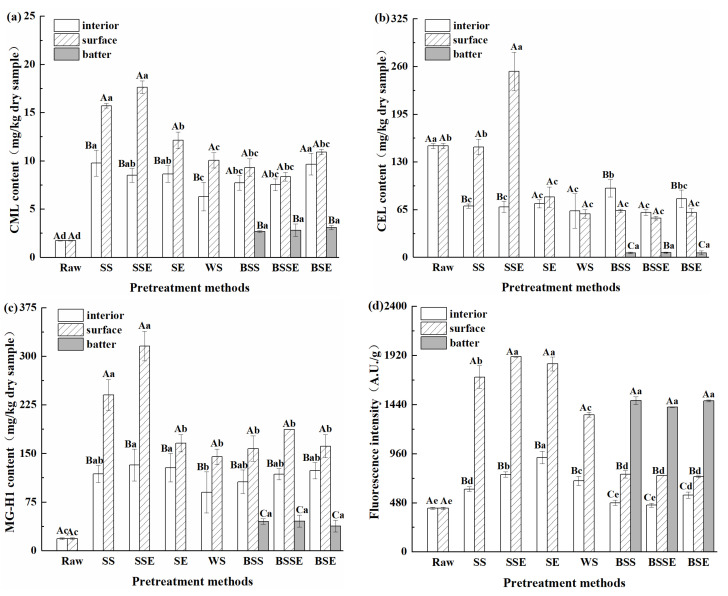
CML (**a**), CEL (**b**), MG-H1 (**c**), and F-AGEs (**d**) contents of fried shrimps under different pretreatment methods (CML, N^ε^-carboxymethyllysine, CEL, N^ε^-carboxyethyllysine, MG-H1, N^ε^-(5-hydro-5-methyl-4-imidazolon-2-yl)-ornithine, Raw, raw shrimps, WS, whole shrimps, SS, shelled shrimps, SSE, shelled shrimps with exscinded back, SE, shrimps with exscinded back, BSS, batter-coated shelled shrimps, BSSE, batter-coated shelled shrimps with exscinded back, BSE, batter-coated shrimps with exscinded back). The data are expressed as mean ± standard deviations of three replicates, different lowercase letters in the same layer indicate significant differences under different pretreatment methods (*p* < 0.05), and different uppercase letters in different layers indicate significant differences under the same pretreatment methods (*p* < 0.05).

AGEs can be divided into fluorescent and non-fluorescent AGEs; CML and CEL are typical lysine-derived non-fluorescent AGEs, and MG-H1 is one of the arginine-derived AGEs [13]. To evaluate the total AGEs levels in fried shrimp, both fluorescent and non-fluorescent AGEs contents were measured simultaneously. Figure 2 and Table 3 show the effect of pretreatment methods on the AGEs contents in the fried shrimps. Overall, the AGEs contents significantly increased (except CEL) in Pacific white shrimp after frying. Notably, the AGEs content in the surface layer of the batter-coated fried shrimps was significantly lower compared to the uncoated groups (*p* < 0.05). As for the non-fluorescent AGEs, the CML and MG-H1 contents in fried shrimps increased by 56.66–936.61% and 129.63–1584.86%, respectively, compared to those in raw samples. And the maximum values of CML (17.62 mg/kg), CEL (253.31 mg/kg), and MG-H1 (315.58 mg/kg) contents were observed in SSE. In addition, the order of CML, CEL, and MG-H1 contents in the surface layer of the uncoated fried shrimps was SSE > SS > SE > WS. However, the non-fluorescent AGEs contents in the surface layer of the batter-coated fried shrimps were close to those of WS. AGEs are mainly produced through the Maillard reaction in the presence of carbonyl and amino compounds [25]. Reducing sugar, lipid oxidation, and sugar autoxidation provide carbonyl compounds, while protein and its oxidative cleavage products provide amino compounds for the Maillard reaction [26]. All the reactions above simultaneously occur during frying and interact with each other via the intermediate products and free radicals [27]. In addition, the frying oil absorbed by samples could promote lipid oxidation, leading to more AGEs formation during frying. It was speculated that the back-exscinded treatment of SSE might enhance lipid oxidation and the Maillard reaction by absorbing oil and oxygen during frying, which generated reactive carbonyl compounds and led to a high AGE contents. For WS, the shrimp shells blocked the internal penetration of oil and heat and further decreased the formation of AGEs during frying. Similarly, the batters also decreased the AGEs content in the surface layer of samples due to their oil and heat resistance. Among three non-fluorescent AGEs, CML and CEL were produced by the reaction of lysine with glyoxal and methylglyoxal, respectively, while MG-H1 was the product of the between arginine and methylglyoxal [26]. MG-H1 content was higher than CML and CEL contents, which might be due to the high arginine content in shrimp. In addition, the CML and MG-H1 contents in fried shrimps were significantly higher than those in raw shrimp, while only the surface layer of SSE had a higher CEL content than that in raw shrimp (Table 3). Similarly, the CEL content also showed a decreasing trend when eggs were cooked at 80 °C for 6 min [28], but further exploration is required to explain the reason for this phenomenon. As for the different layers in fried shrimps, the AGEs contents in the interior layer of samples were lower than those in the surface layer, which might be because the low temperature (<100 °C) in the interior layer weakened the Maillard reaction and lipid oxidation during frying. The batter layer had the lowest non-fluorescent AGEs contents, which might be related to the lower protein content in the batter layer decreasing the precursor of AGEs formation compared with the other layers.

As illustrated in Figure 2d and Table 3, the F-AGEs contents in the surface layer of the fried shrimps significantly increased after frying. The WS had the lowest F-AGEs contents among the uncoated groups, while the SSE had the highest F-AGEs contents. This suggested that the generation of F-AGEs was depressed by shrimp shells but promoted by the back-exscinded treatment. In contrast, the F-AGEs contents on the surface layer of batter-coated fried shrimps were lower than that of WS, which was consistent with the result of non-fluorescent AGEs. However, the order of F-AGEs contents in the different layers of the batter-coated fried shrimps was batter layer > surface layer > interior layer, which was different from that of non-fluorescent AGEs. In this work, non-fluorescent AGEs are single amino acid modified products, but F-AGEs are cross-linking products made of multiple amino acids with dicarbonyl compounds [26]. Therefore, it is speculated that the different formation pathway of fluorescent and non-fluorescent AGEs is one of the reasons for the different order. In addition, the determination of fluorescence spectroscopy is not specific, and the influence of starch may lead to a higher fluorescence intensity in the batter layer, which was similar to a previous study in that samples coated with wheat flour had a higher fluorescence intensity than uncoated samples [7]. In sum, the pretreatment methods significantly affect the AGEs levels in the fried shrimps. Compared with the AGEs content in fried fish products in previous studies [7,24,29], a higher level of AGEs was observed in fried shrimps. Therefore, it is essential to understand and control the different AGEs in fried shrimp. To further reveal the plausible mechanism underlying the formation of AGEs in fried shrimp, the basic compositions and physicochemical Indicators in the fried samples were analyzed.

### 3.2. Analysis of Water, Oil, and Protein Contents

**Figure 3:**
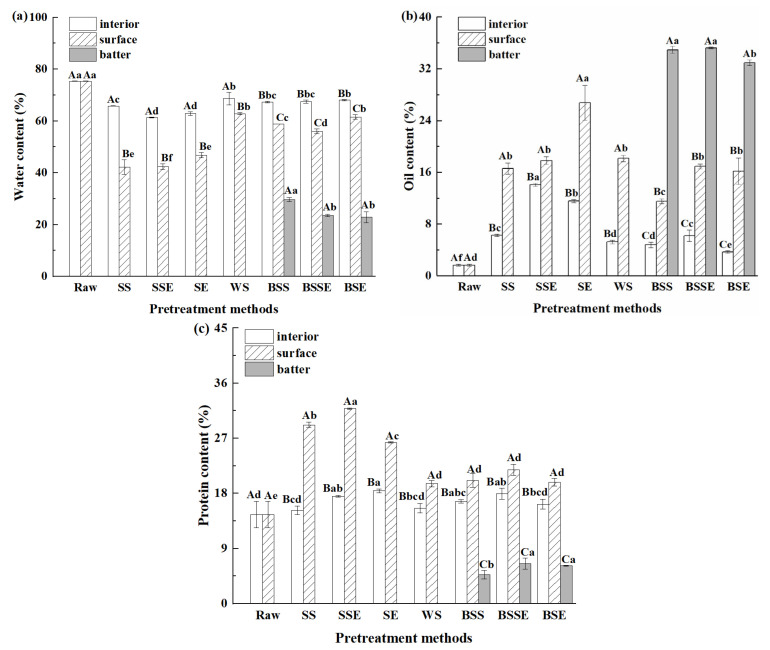
Water (**a**), oil (**b**), and protein (**c**) contents of fried shrimps under different pretreatment methods. Raw, raw shrimps, WS, whole shrimps, SS, shelled shrimps, SSE, shelled shrimps with exscinded back, SE, shrimps with exscinded back, BSS, batter-coated shelled shrimps, BSSE, batter-coated shelled shrimps with exscinded back, BSE, batter-coated shrimps with exscinded back. The data are expressed as mean ± standard deviations of three replicates, different lowercase letters in the same layer indicate significant differences under different pretreatment methods (*p* < 0.05), and different uppercase letters in different layers indicate significant differences under the same pretreatment methods (*p* < 0.05).

The basic compositions of food provide precursors for the physicochemical reactions during processing. Figure 3 illustrates the changes in the basic compositions of the fried shrimps. The water content significantly decreased, while the oil, and protein contents significantly increased after frying (*p* < 0.05). Overall, the decrease in water content was accompanied by an increase in oil and protein contents. The orders of water, oil, and protein contents on the surface layer of four uncoated groups were WS > SE > SS > SSE, SE > SSE ≈ SS ≈ WS, and SS ≈ SSE > SE > WS, respectively. As for SSE, it is possible that the back-exscinded treatment raised the oil absorption and water evaporation of the fried shrimps by increasing the contact area between the shrimps and the frying oil during frying. On the other hand, protein content significantly increased due to water loss. Thus, SSE had a higher protein content than that the other samples, while WS had the lowest protein content. It was suggested that water evaporation provides void spaces for oil absorption [4], and frying oil enters the interior of fried foods through tiny pores under pressure [30]. Therefore, water loss is usually accompanied by oil absorption during frying, which is one of the reasons SSE had a higher oil content and lower water content than SS, and WS had the highest water content and oil absorption. However, high water and oil contents were found in SE, and it was reported that oil absorption was related to the equilibrium between the adhesion and drainage of oil in fried foods [17]. Thus, it was presumed that the shrimp shells inhibited the evaporation of water during frying and the drainage of oil during cooling in SE, which caused frying oil to be left in the gap between the shells and the surface of the shrimp during frying. As for the surface of the batter-coated fried shrimps, the orders of water and oil contents were BSE > BSS > BSSE and BSE > BSSE ≈ BSS, respectively. And there was no significant difference in the protein content (*p* > 0.05). The water content in the surface layer of the batter-coated samples was lower than that in WS. In addition, the oil content was similar to that in WS, which indicated that the formation of a crispy coating on the batter-coated samples reduced the water loss and oil absorption of samples. However, the oil content in the batter layer was significantly higher than that in other layers, while the water and protein contents were the opposite. This might be because the batter layer mainly consisted of batter and breadcrumbs, and the formation of a crispy crust provided porous channels for oil absorption. In addition, the increasing roughness of the surface layer in samples with the batter-coating treatment was another reason for the high oil uptake. The low protein content in the batter layer originated from the significant difference between the composition of the shrimps and the batter layer. Previous studies reported that oil and protein levels had a positive correlation with the level of AGEs, while the water level was negatively correlated with the level of AGEs [9]. Similarly, combined with the AGEs contents in uncoated samples (Figure 2), the sample with noticeable changes in basic compositions (low water content, high oil and protein content) had high AGEs contents, which indicated that the basic compositions were closely related to the formation of AGEs.

### 3.3. Analysis of Lipid and Protein Oxidation

**Figure 4:**
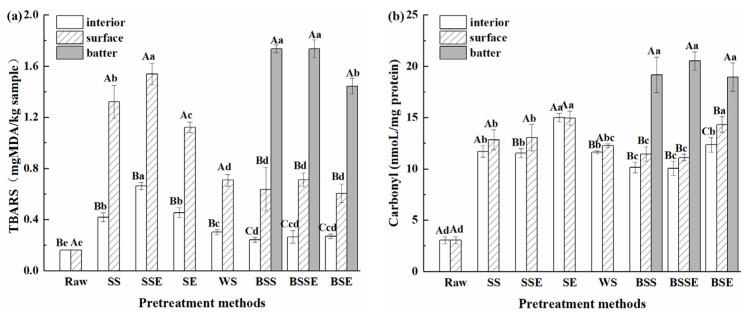
Lipid oxidation (**a**) and protein oxidation (**b**) of fried shrimps under different pretreatment methods (Raw, raw shrimps, WS, whole shrimps, SS, shelled shrimps, SSE, shelled shrimps with exscinded back, SE, shrimps with exscinded back, BSS, batter-coated shelled shrimps, BSSE, batter-coated shelled shrimps with exscinded back, BSE, batter-coated shrimps with exscinded back). The data are expressed as mean ± standard deviations of three replicates. Different lowercase letters in the same layer indicate significant differences under different pretreatment methods (*p* < 0.05), and different uppercase letters in different layers indicate significant differences under the same pretreatment methods (*p* < 0.05).

The TBARS value is widely used to assess the lipid oxidation degree of food. As shown in Figure 4a, the TBARS values significantly increased after frying (*p* < 0.05). The TBARS values were ranked as SSE > SS > SE > WS in the surface layer of four uncoated samples. Generally, high temperatures and oxygen promoted lipid oxidation [31]. Therefore, it was assumed that the highest TBARS value in SSE was because the back-exscinded treatment promoted lipid oxidation by absorbing frying oil and increasing the contact area between samples and frying oil or oxygen. On the other hand, the shells decreased the TBARS value by preventing the penetration of oil and heat transfer in SE and WS during frying. Similarly, lower TBARSs values were obtained in the surface layer of batter-coated samples for the protection of the batter layer. Additionally, no significant difference was observed between the surface layer of batter-coated samples and WS (*p* > 0.05), and the TBARSs value in the interior layer of batter-coated samples was even lower than that of WS. As for different sample layers, the TBARSs value order was batter layer > surface layer > interior layer. It was hypothesized that the higher TBARSs values in the batter layer of samples were related to the high oil content (Figure 3b) and temperature. On the contrary, the low TBARSs values in the interior layer of samples were due to the low oil content (Figure 3b) and temperature. Combined with the results of AGEs contents (Figure 2), high AGEs contents were present in the uncoated treatment group with high TBARS values (especially in SSE), which indicated that the formation of AGEs was promoted by lipid oxidation.

Protein carbonylation is one of the remarkable phenomena in protein oxidation, which can be reflected by the carbonyl content. As shown in Figure 4b, the carbonyl content significantly increased after frying (*p* < 0.05), especially in the batter layer of samples, and the surface and interior layer of samples showed a similar protein carbonyl content. Previous studies have reported that the carbonyl groups generated by lipid oxidation can react with 2,4-dinitrophenylhydrazine for a color reaction [32]. Combined with the high TBARS value in the batter layer (Figure 4a), it was speculated that carbonyl compounds generated by lipid oxidation were the main reason for the high carbonyl content in the batter layer of samples. However, the order of carbonyl content was SE > SSE ≈ SS ≈ WS in the uncoated samples, which showed that SSE and SS had higher lipid oxidation and AGEs contents but a lower carbonyl content than SE. Estévez et al. [33] reported that some carbonyl compounds (such as a-aminoadipic and c-glutamic semialdehydes) might further react with free amino acids, which might be responsible for the formation of Strecker aldehydes and AGEs, causing a reduction in the protein carbonyl content. Therefore, it was assumed that the high temperature and lipid oxidation in SSE and SS provided intense oxidation conditions during frying, promoting the combination of protein carbonyl compounds and free amino acids. In addition, except for protein carbonylation, the formation of intra- and intermolecular cross-links, the loss of sulfhydryl groups, and tryptophan fluorescence are vital manifestations of protein oxidation [34,35]; there might be multiple chemical manifestations of protein oxidation and low carbonyl content in SSE and SS. As for the WS, similar to TBARSs, the shells prevented the penetration of oil and heat transfer in SE and further weakened the protein oxidation.

### 3.4. Analysis of Free Amino Groups

**Figure 5:**
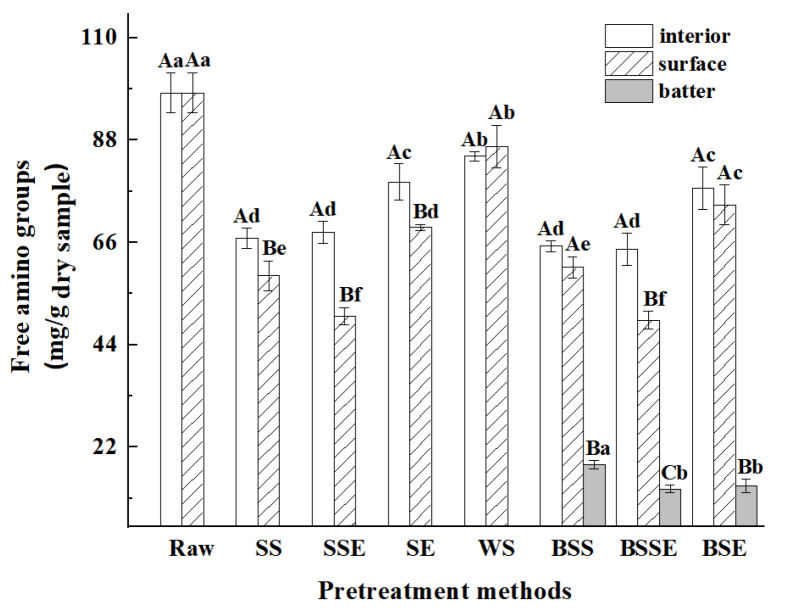
Free amino groups in fried shrimps under different pretreatment methods (Raw, raw shrimps, WS, whole shrimps, SS, shelled shrimps, SSE, shelled shrimps with exscinded back, SE, shrimps with exscinded back, BSS, batter-coated shelled shrimps, BSSE, batter-coated shelled shrimps with exscinded back, BSE, batter-coated shrimps with exscinded back). The data are expressed as the mean ± standard deviations of three replicates. Different lowercase letters in the same layer indicate significant differences under different pretreatment methods (*p* < 0.05), and different uppercase letters in different layers indicate significant differences under the same pretreatment methods (*p* < 0.05).

Free amino groups can participate in reactions reducing sugars and dicarbonyl compounds, thereby facilitating the formation of advanced glycation end products (AGEs) through the Maillard reaction [26]. Thus, the levels of free amino groups can be used to evaluate the degree of protein glycation in samples [24]. As shown in Figure 5, the amounts of free amino groups in samples significantly decreased after frying (*p* < 0.05). Similarly, Han et al. [28] pointed out that all fried sturgeon patties had lower free amino groups than those in raw sturgeon. As for the uncoated samples, the order of free amino groups was WS > SE > SS > SSE, and there was a consistent tendency on the surface layer of corresponding batter-coated fried shrimps. Amino acids in protein could be modified by dicarbonyl compounds from the Maillard reaction and lipid oxidation, leading to the formation of AGEs [15]. As for the different pretreatments, combined with the TBARS values (Figure 4a), it was assumed that the high dicarbonyl compounds from lipid oxidation in SSE consumed more free amino groups than those in the other samples. On the contrary, samples with shells (SS and SSE) might reduce the consumption of free amino groups. Thus, the WS had the highest free amino groups. In addition, combined with the AGEs contents in Figure 2, the samples with high AGEs contents also had low free amino groups, which indicated that the decreased free amino groups participated in the generation of AGEs during frying. As for different layers of samples, the amounts of free amino groups on the surface layer were lower than those in the interior layer, while the batter layer had the lowest free amino groups. It might be the low-temperature depressed protein glycation in the interior layer during frying. The low AGEs and free amino contents were obtained in the batter layer of samples, the low protein content of the batter layer (Figure 3a) might have led to a limitation of the generation of AGEs.

### 3.5. Analysis of Browning Intensity

**Figure 6:**
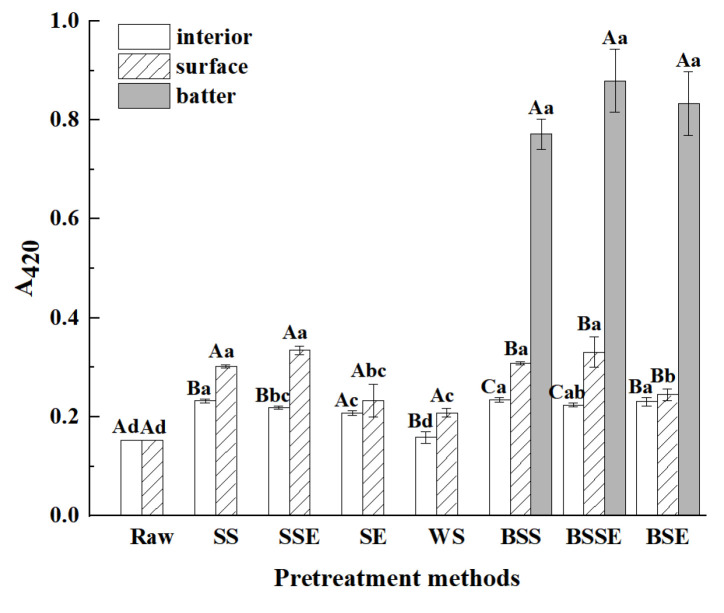
Browning intensity of fried shrimps under different pretreatment methods (Raw, raw shrimps, WS, whole shrimps, SS, shelled shrimps, SSE, shelled shrimps with exscinded back, SE, shrimps with exscinded back, BSS, batter-coated shelled shrimps, BSSE, batter-coated shelled shrimps with exscinded back, BSE, batter-coated shrimps with exscinded back). The data are expressed as mean ± standard deviations of three replicates. Different lowercase letters in the same layer indicate significant differences under different pretreatment methods (*p* < 0.05), and different uppercase letters in different layers indicate significant differences under the same pretreatment methods (*p* < 0.05).

The browning intensity of the samples (absorbance at 420 nm, A_420_) was positively correlated with the content of melanoidin, a product of the Maillard reaction [36]. As shown in Figure 6, frying significantly elevated the A_420_ in fried shrimps (*p* < 0.05). In the uncoated fried shrimps, the order of browning intensity was SSE > SS > SE > WS. And the value of A_420_ in the interior of WS was similar to that of raw shrimp. It was reported that high temperature and low water content could promote non-enzymatic browning reactions [25]. In addition, the Maillard reaction and lipid oxidation are closely related to the quality of final products, and they can also interact with each other by the intermediates [33]. According to the changes in water content (Figure 3a) and TBARS values (Figure 4a), it was hypothesized that the back-exscinded treatment promoted the Maillard reaction by elevating the lipid oxidation and providing a high-temperature and low-water environment, while the shells weakened the physicochemical reactions by providing a barrier. As for different layers of samples, the order of browning intensity was batter layer > surface layer > interior layer. It was speculated that the batter layer in the batter-coated fried shrimps enriched the precursors of Maillard and caramelization reaction by providing more carbohydrates. Thus, the batter layer had a high A_420_. As for the interior layer, the low A_420_ might be because the low temperature depressed chemical reactions during frying.

### 3.6. Analysis of Color

The color of fried foods has a vital effect on the organoleptic acceptance and purchase desire of consumers, and it is also closely related to the Maillard reaction. Compared with the color of raw shrimp, a marked increase in the L* and b* values of fried shrimps were found (*p* < 0.05), while the a* value only significantly increased on the surface layer of the fried shrimps, as shown in Table 4 (*p* < 0.05). As for the surface layer of the samples, it was speculated that the Maillard reaction and the alterations in oil and astaxanthin mainly influenced the variations of color. Usually, the bluish-brown color in raw shrimp originates from bound-state between astaxanthin with protein, while the reddish-orange in cooked shrimps might be due to free astaxanthin escaping from protein denaturation during heating [2,37]. In addition, astaxanthin is highly susceptible to oxidation and isomerization of its instability and oil solubility during frying [38], which leads to the loss of astaxanthin and low a* and b* values. As for the uncoated samples, the low a* value in SSE might have been related to the loss and transformation of astaxanthin caused by intense oxidative reaction (Figure 4a). In contrast, the higher a* and b* values in BSE, SE, and WS might be due to the high content of astaxanthin in shrimp shells. Additionally, the interior layer of samples had a higher L* value and lower a* and b* values than the surface layer. It was reported that L* value had a negative correlation with the content of melanoidin [39]. Combined with the results of A_420_ in the batter layer of the batter-coated samples (Figure 6), it was hypothesized that the accumulation of melatonin caused the lower L* value. In addition, the oil absorption and water loss could decrease the L* value by weakening the light reflection [17], which might be a reason for the decrease in L* value in samples during frying. As for the interior layer of samples, the higher L* value and lower b* value were present in the samples with batter coating or shells (BSS, BSSE, BSE, SE, WS), which might be attributed to the weak physicochemical changes caused by the protection of batter coating and shells. On the contrary, the back-exscinded treatment in the SSE possibly promoted the Maillard reaction, lipid oxidation, and frying oil penetration, causing the lowest L* value and the highest b* value.

### 3.7. Correlation Analysis

**Figure 7:**
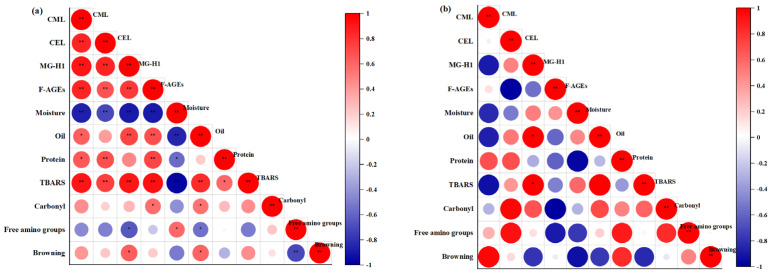

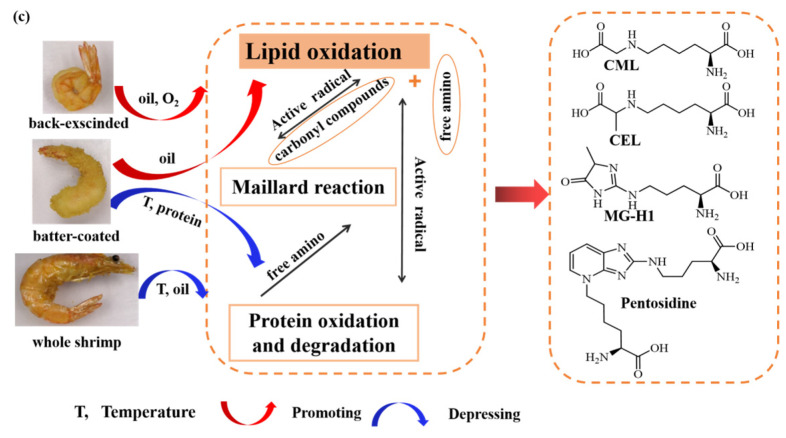
Correlations of AGEs and physicochemical properties in the non-batter (**a**) and batter layer (**b**) of fried shrimps, and the formation of AGEs in fried shrimps under different pretreatment methods (**c**). F-AGEs, Fluorescent AGEs, * Significant correlation *p* < 0.05, ** Significant correlation *p* < 0.01.

To clarify the decisive reaction influencing the generation of AGEs in fried shrimps, we analyzed the correlations between the generation of AGEs and the physicochemical indicators of fried shrimps. Considering the apparent difference between basic compositions in batter and non-batter layers (Figure 3), the correlation analyses were performed separately. As shown in Figure 7, the correlation analysis presented a significant difference between the batter and non-batter layers. As for the batter layer, only MG-H1 content had significant positive correlations with oil content and TBARS value (*p* < 0.05). In contrast, there were more significant correlations between other indicators and AGEs contents (*p* < 0.05) in the non-batter layer of fried shrimps. Therefore, the correlation analysis was mainly focused on the non-batter layer in the fried shrimps. To be specific, correlations between TBARS value and AGEs contents were positive and significant (*p* < 0.01), but browning intensity only had a significant positive correlation with MG-H1 (*p* < 0.05). Moreover, the correlation coefficients between the TBARSs value and the AGEs contents were the highest (0.909, 0.747, 0.919, and 0.928 for CML, CEL, MG-H1, and F-AGEs). Hence, the production of AGEs in fried shrimps was influenced by both the Maillard reaction and lipid oxidation. Notably, lipid oxidation played a more significant role in AGEs formation compared to the Maillard reaction. While AGEs are primarily formed through the Maillard reaction, another important pathway involves the reaction between α-dicarbonyl compounds (such as glyoxal and methylglyoxal) generated from oxidative reactions and amino groups [25,26]. Lipid oxidation produces numerous free radicals and hydroperoxides at the initial stage. As a result, some lysine or arginine might be exposed to the oxidation and degradation of proteins under the influence of free radicals [40,41]. On the other hand, hydroperoxides are susceptible to secondary oxidation products, providing more α-dicarbonyl compounds [40]. Consequently, lipid oxidation contributes to the formation of AGEs by facilitating both the Maillard reaction and the production of α-dicarbonyl compounds. Generally, the reaction between free amino groups and carbonyl compounds could participate in the Maillard reaction during thermal processing [42]. The results of correlation analysis were consistent with the above reactions in this work. For example, negative correlations were found among free amino groups, browning intensity, and AGEs contents. Moreover, the level of TBARS value exhibited a positive correlation with both the intensity of browning and the carbonyl content. This indicated that oxidation and Maillard reactions simultaneously occurred and promoted each other during frying. As shown in Figure 7a, the basic compositions of fried shrimps were correlated with the formation of AGEs. To be specific, water content had a significant negative correlation with AGEs contents (*p* < 0.01), while oil content and protein content had significant positive correlations with AGEs contents (*p* < 0.05). Specifically, water content had a significant negative correlation with AGEs contents (*p* < 0.01), which might be because low water content promoted the Maillard reaction. However, oil and protein content had significant positive correlations with AGEs contents (*p* < 0.05). It was possible that the absorption of frying oil and high protein content promoted lipid oxidation and the Maillard reaction during frying, which increased AGEs contents. In addition, the correlations of oil content, protein content, and browning intensity with water content were significantly negative (*p* < 0.05). However, oil content positively correlated with the TBARS value (*p* < 0.01). This suggested that the high temperature promoted the evaporation of water and elevated oil and protein content in fried shrimps. The increased oil content could encourage the production of AGEs by boosting oxidative reaction and providing reactive carbonyl products. On the other hand, it also provided a conducive condition for the Maillard reaction during frying. Therefore, the changes in chemical reactions and basic compositions controlled the formation of AGEs, and lipid oxidation was the decisive factor of AGEs contents in the fried shrimps.

In summary, the possible formation mechanism of AGEs in fried shrimp was proposed as shown in Figure 7c. The pretreatment methods altered the basic compositions and frying environment of samples, which significantly affected the chemical reactions and further influenced the formation of AGEs. To be specific, the samples with back-exscinded treatment brought oxygen and frying oil, which increased AGEs contents by promoting lipid oxidation and the Maillard reaction during frying. As for the samples with the batter-coating treatment, although the absorption of frying oil promoted lipid oxidation in the batter layer, the battering limited the generation of AGEs by decreasing protein content and free amino groups, the important precursors of the Maillard reaction. The battering also reduced the temperature and oil penetration in the surface and interior layers, which depressed the physicochemical reactions and the formation of AGEs. Similarly, the shells of the whole shrimps inhibited the reactions by depressed oil penetration and heat transfer during frying, which explained the low AGEs content in WS. Among the chemical reactions involved in frying processing, lipid oxidation might promote the production of AGEs by generating free radicals and reactive carbonyl groups, which could contribute to the protein oxidative degradation and Maillard reaction, respectively. Combined with the results of correlation analysis, lipid oxidation (the decisive chemical reaction) might promote the formation of AGEs by providing carbonyl compounds and free amino groups from protein degradation.

## 4. Conclusions

The pretreatment methods significantly affect the physicochemical indicators and AGEs contents in fried shrimp. The highest AGEs contents and changes in physicochemical indicators were observed in SSE. Although the batter-coated pretreatment significantly reduced the AGEs contents of fried shrimps, it increased the oil content in the batter layer of samples. The results suggested that WS was the optimal pretreatment for its lowest AGEs and oil content, and desirable color (high L* and a* values). Correlation analysis indicated that the critical reaction promoting the formation of AGEs was lipid oxidation, which provided carbonyl compounds and enhanced the combination of free amino groups and α-dicarbonyl compounds in fried shrimps. Therefore, it is possible to inhibit the AGEs content of fried shrimps by diminishing the particular chemical reaction (lipid oxidation) in future studies.

## Figures and Tables

**Table 1:** Elution gradient of CML, CEL, and MG-H1.

Time (min)	0	1	6.5	9	10
A	15%	15%	35%	35%	15%
B	85%	85%	65%	65%	85%

Solvent A, 5 mM ammonium acetate and 0.1% formic acid, solvent B, 100% acetonitrile.

**Table 2:** Mass spectrometry parameters for CML, CEL, and MG-H1.

Analyte	Precursor Ion (m/z)	Product Ion (m/z)	Collision Energy (eV)	Cone (V)	Collision (V)
CML	205	130	20	20	15
		84 *	30	20	20
CEL	219	130	20	20	15
		84 *	30	20	20
MG-H1	229	114	20	20	15
		70 *	30	20	20

Product ion with an asterisk (*) indicates quantitative ion.

**Table 3:** The change rate of AGEs.

Pretreatment Methods		Change Rate of CML/%	Change Rate of CEL/%	Change Rate of MG-H1/%	Change Rate of F-AGEs/%
Surface layer	SS	851.83 ± 48.52 ^a^	−1.49 ± 0.86 ^b^	1248.41 ± 146.06 ^b^	298.74 ± 26.15 ^b^
	SSE	936.61 ± 38.92 ^a^	65.95 ± 17.19 ^a^	1584.86 ± 121.57 ^a^	344.89 ± 1.11 ^a^
	SE	613.05 ± 50.78 ^b^	−51.4 ± 2.15 ^c^	786.05 ± 71.03 ^cd^	328.57 ± 15.77 ^a^
	WS	491.44 ± 47.15 ^cd^	−61.3 ± 3.91 ^c^	672.81 ± 64.55 ^d^	212.82 ± 5.18 ^c^
	BSS	447.39 ± 53.27 ^cd^	−58.26 ± 1.34 ^c^	740.7 ± 104.5 ^d^	76.71 ± 8.46 ^d^
	BSSE	433.77 ± 75.57 ^d^	−61.37 ± 6.49 ^c^	946.68 ± 82.45 ^c^	74.07 ± 0.38 ^d^
	BSE	542.22 ± 15.63 ^bc^	−59.89 ± 3.24 ^c^	761.46 ± 94.66 ^cd^	71.43 ± 1.97 ^d^
Interior layer	SS	474.02 ± 79.4 ^a^	−54.29 ± 1.91 ^bc^	532.32 ± 71.63 ^a^	43.07 ± 5.19 ^c^
	SSE	399.94 ± 41.44 ^ab^	−54.91 ± 4.86 ^bc^	605.53 ± 131.76 ^a^	76.29 ± 5.95 ^b^
	SE	408.36 ± 51.45 ^ab^	−52.19 ± 3.55 ^bc^	583.8 ± 117.11 ^a^	115.4 ± 14.23 ^a^
	WS	320.37 ± 15.39 ^b^	−50.17 ± 7.3 ^b^	478.77 ± 4.5 ^a^	61.82 ± 10.12 ^b^
	BSS	354.45 ± 44.99 ^b^	−42.8 ± 0.2 ^a^	467.06 ± 97.84 ^a^	15.34 ± 0.57 ^cd^
	BSSE	342.42 ± 36.74 ^b^	−60.05 ± 2.57 ^c^	530.97 ± 46.67 ^a^	8.79 ± 2.67 ^d^
	BSE	468.07 ± 65.36 ^a^	−52.23 ± 0.59 ^bc^	559.46 ± 67.38 ^a^	29.36 ± 6.96 ^cd^
Interior layer	BSS	56.66 ± 5.57 ^b^	−95.97 ± 0.63 ^a^	139.64 ± 22.68 ^a^	76.71 ± 8.46 ^a^
	BSSE	95.1 ± 4.09 ^a^	−96.52 ± 1.25 ^a^	169.82 ± 11.25 ^a^	74.07 ± 0.38 ^a^
	BSE	87.62 ± 9.28 ^a^	−95.96 ± 1.78 ^a^	129.63 ± 11.75 ^a^	71.43 ± 1.97 ^a^

The data are expressed as mean ± standard deviations of three replicates. Different lowercase letters in the same column indicate significant differences under different pretreatment methods (*p* < 0.05). Raw, raw shrimps, WS, whole shrimps, SS, shelled shrimps, SSE, shelled shrimps with exscinded back, SE, shrimps with exscinded back, BSS, batter-coated shelled shrimps, BSSE, batter-coated shelled shrimps with exscinded back, BSE, batter-coated shrimps with exscinded back.

**Table 4:** The color of fried shrimps under different pretreatment methods.

Pretreatment Methods		L*	a*	b*
Surface/batter layer	Raw	45.15 ± 0.93 ^e^	−1.01 ± 0.2 ^e^	1.63 ± 0.58 ^e^
	SS	69.38 ± 1.04 ^b^	6.03 ± 1.02 ^b^	25.13 ± 1.35 ^c^
	SSE	65.79 ± 1.97 ^c^	5.62 ± 1.13 ^b^	26.53 ± 2.28 ^c^
	SE	69.13 ± 1.06 ^b^	7.63 ± 1.89 ^a^	34.11 ± 2.7 ^a^
	WS	72.6 ± 1.19 ^a^	7.55 ± 1.43 ^a^	29.37 ± 3.33 ^b^
	BSS	63.05 ± 1.69 ^d^	2.59 ± 0.47 ^d^	22.37 ± 1.4 ^d^
	BSSE	62.22 ± 1.36 ^d^	1.92 ± 0.54 ^d^	21.84 ± 1.18 ^d^
	BSE	62.18 ± 1.76 ^d^	4.25 ± 0.55 ^c^	22.42 ± 0.95 ^d^
Interior layer	Raw	45.15 ± 0.93 ^e^	−1.01 ± 0.2 ^ab^	1.63 ± 0.58 ^c^
	SS	79.76 ± 1.57 ^cd^	−0.72 ± 0.28 ^ab^	6.01 ± 1.27 ^b^
	SSE	78.06 ± 2.23 ^d^	0.07 ± 0.01 ^a^	10.58 ± 3.07 ^a^
	SE	82.04 ± 1.05 ^bc^	−1.44 ± 0.14 ^b^	10.03 ± 0.7 ^a^
	WS	83.87 ± 3.17 ^ab^	−0.63 ± 0.07 ^ab^	7.58 ± 1.96 ^a^
	BSS	85.92 ± 0.94 ^a^	−1.03 ± 0.23 ^ab^	7.2 ± 1.69 ^b^
	BSSE	82.53 ± 1.83 ^b^	−0.29 ± 0.08 ^ab^	5.17 ± 0.63 ^b^
	BSE	84.54 ± 0.56 ^ab^	−1.11 ± 0.18 ^ab^	5.92 ± 0.33 ^b^

The data are expressed as the mean ± standard deviations of eight replicates. Different lowercase letters in the same column indicate significant differences under different pretreatment methods (*p* < 0.05). Raw, raw shrimps, WS, whole shrimps, SS, shelled shrimps, SSE, shelled shrimps with exscinded back, SE, shrimps with exscinded back, BSS, batter-coated shelled shrimp, BSSE, batter-coated shelled shrimps with exscinded back, BSE, batter-coated shrimps with exscinded back.

## Data Availability

Data are contained within the article.

## Abbreviations

AGEs, advanced glycation end products; CML, N^ε^-carboxymethyllysine; CEL, N^ε^-carboxyethyllysine; MG-H1, N^ε^-(5-hydro-5-methyl-4-imidazolon-2-yl)-ornithine; TBARS, thiobarbituric acid reactive substances; F-AGEs, Fluorescent AGEs.
